# NHL Pathological Image Classification Based on Hierarchical Local Information and GoogLeNet-Based Representations

**DOI:** 10.1155/2019/1065652

**Published:** 2019-03-21

**Authors:** Jie Bai, Huiyan Jiang, Siqi Li, Xiaoqi Ma

**Affiliations:** ^1^Northeastern University, Shenyang 110819, China; ^2^Software College, Northeastern University, Shenyang 110819, China; ^3^School of Science and Technology, Nottingham Trent University, Nottingham, UK

## Abstract

**Background:**

Accurate classification for different non-Hodgkin lymphomas (NHL) is one of the main challenges in clinical pathological diagnosis due to its intrinsic complexity. Therefore, this paper proposes an effective classification model for three types of NHL pathological images, including mantle cell lymphoma (MCL), follicular lymphoma (FL), and chronic lymphocytic leukemia (CLL).

**Methods:**

There are three main parts with respect to our model. First, NHL pathological images stained by hematoxylin and eosin (H&E) are transferred into blue ratio (BR) and Lab spaces, respectively. Then specific patch-level textural and statistical features are extracted from BR images and color features are obtained from Lab images both using a hierarchical way, yielding a set of hand-crafted representations corresponding to different image spaces. A random forest classifier is subsequently trained for patch-level classification. Second, H&E images are cropped and fed into a pretrained google inception net (GoogLeNet) for learning high-level representations and a softmax classifier is used for patch-level classification. Finally, three image-level classification strategies based on patch-level results are discussed including a novel method for calculating the weighted sum of patch results. Different classification results are fused at both feature 1 and image levels to obtain a more satisfactory result.

**Results:**

The proposed model is evaluated on a public IICBU Malignant Lymphoma Dataset and achieves an improved overall accuracy of 0.991 and area under the receiver operating characteristic curve of 0.998.

**Conclusion:**

The experimentations demonstrate the significantly increased classification performance of the proposed model, indicating that it is a suitable classification approach for NHL pathological images.

## 1. Introduction

Lymphoma is a type of malignant tumors that originate from the lymphoid hematopoietic tissues [1], which are divided into the Hodgkin lymphomas (HL) and the Non-Hodgkin's lymphomas (NHL). NHL is one of the common malignant tumors with the fastest growth rate, and chronic lymphocytic leukemia (CLL), follicular lymphoma (FL), and mantle cell lymphoma (MCL) are the major types of NHL. Pathological characteristics vary complicatedly among different lymphoma subtypes. And the vision disparities of different subtypes are indistinguishable. Therefore, accurate classification of various lymphoma histopathology has become one of the difficult challenges for hematologists and pathologists, as well as the development of computer vision methods.

Histopathological image analysis is an important part of computer aided diagnosis (CAD). Complex structure information in pathological images provides great value for diagnosis of many diseases including most tumors. There is a mass of complicated pathological information including abundant spatial structure, various cell types, and variation in cell morphology in histopathological images. Traditionally, these characteristics are found and analyzed by pathologists with clinical experiences, which is time-consuming and inefficient. With the development of CAD, approaches have been designed to automatically extract expressive features from histopathological images for subsequent differentiation tasks and then to facilitate disease diagnosis.

Traditionally, there are a large number of feature extraction and classification approaches based on traditional machine learning or handcrafted features which reflect low level characteristics involving morphological descriptors, spatial arrangement, color and texture features. Sertel* et al.* [2] obtained semantic description by model-based intermediate representation (MBIR) and combined MBIR with low level texture features for the grading of the follicular lymphoma (FL). However, those three grades of FL have significant morphological differences which make it easier to identify them. Baker* et al.* [3] utilized a coarse-to-fine method to extract the local features in a two-stage way for brain tumor whole slide classification, but they also pointed out that the method might not be efficient for small pathological image classification with identified lesions. There are some approaches with good classification performances but just for binary classification tasks. Fukuma* et al.* [4] extracted morphological features and spatial arrangement features based on graph theory as feature descriptions. Cao* et al.* [5] combined texture features, spatial distribution, and semantic features of the nuclear structure to describe the various morphology of the nucleus and the structural and interpretation information of the image. These binary classification methods are hard to be extended to multiclass classification. Zhou* et al.* [6] developed an image classification platform to give a standard solution for different problems, which sacrificed pertinence for specific disease and resulted in a low accuracy of 70.9% for lymphoma classification. Meng* et al.* [7] developed an approach to lymphoma classification that divided images into 25 subblocks from which a set of 505 color and texture features were extracted. However, its weighting scheme was performed in a class level and majority voting was still used for image label acquisition, which might lead to losing a part of local information. Song* et al.* [8] proposed a method to extract high-dimensional multimodal descriptor, and subcategory discriminant transform (SDT) was used to enhance discriminative power of descriptors. This approach extracted sufficient image information and achieved decent results, but it used deep learning method just for comparison purpose instead of benefitting from making use of the full power of deep learning. Those above approaches extract customized, manually designed features which require domain-specific knowledge, and the extracted features just represent a portion of the image characteristics.

With the rapid development of the theory of deep learning, automatic learning approaches are developed for feature extraction and medical image classification. Zhou* et al.* [9] proposed a multispectral feature learning model based on convolutional sparse coding (CSC) and spatial pyramid matching (SPM), which could automatically learn a set of convolution filter banks from separate spectra. Vu* et al.* [10] proposed an automatic feature extraction framework by learning class-specific dictionaries, and a low-complexity method was used for histopathology classification and disease grading. They tried to explore the histopathological image classification issue from the perspective of feature discovery and dictionary learning and obtained promising results in different datasets. Approaches based on convolutional neural network (CNN) have been widely applied in various medical visual recognition tasks and demonstrated promising performances [11]. Gao* et al.* [12] proposed an automatic framework for HEp-2 cell image classification, where a CNN with three convolutional layers is employed. The recently prevailed various CNN pretrained on ImageNet can be explored to extend this work. Sirinukunwattana* et al.* [13] proposed spatially constrained convolutional neural network (SC-CNN) for nucleus detection and a novel neighboring ensemble predictor (NEP) was developed to more accurately predict the cell nuclei class. Note that those methods based on deep learning are awfully time consuming since they need the training process.

Recently, there are some approaches that combine traditional handcrafted features and high-level representations of deep learning methods. Codella* et al.* [14] extracted a combination of local binary pattern (LBP) and CNN features based on several enhanced and segmented results, so it required an early precise segmentation process. Song* et al.* [15] proposed Fisher vector (FV) encoding combined with multiple types of local features including high-level features from CNN and applied a novel separation-guided dimension reduction method, which achieved state-of-the-art results. However, it had low feature extraction efficiency due to complicated computation.

To sum up, while a number of approaches have been proposed, accurate histopathological image classification, especially for malignant lymphoma images, still remains a challenging task due to some issues. First, the key to classification task is feature extraction and the above methods all explored different features to represent image information, but more efficient and complete representations still need extracting in an appropriate way. Second, some methods performed classification by employing patches-division and showed their ability for local knowledge extraction, but they have not considered different classification contributions for those cropped patches with diverse locations in one image. Third, most of those methods were just based on either handcrafted features or high-level features. Some approaches combined them together, but still needed improvement to achieve better performance with lower computational complexity.

In this study, to address these problems, we propose a hierarchical local information and transfer learning based approach specific to transformed image spaces for the classification of MCL, FL, and CLL histopathological images. Original RGB images are transformed to blue ratio and CIE Lab image space [16] to amplify the texture and color information, respectively. Then image patches are cropped at two hierarchies; parent patches are cropped from transformed images and subpatches are obtained from parent patches by utilizing sliding window. Handcrafted features are extracted from subpatches specific to different image spaces and parent-patch features are statistically computed from corresponding subpatches representations. Random forest classifier is trained for parent patch classification. Different strategies for image level classification are discussed, including a novel distance matrix weighting (*DMW*) method for calculating weighted sum of parent patch results. Furthermore, a pretrained Google Inception Net v3 model is fine-tuned to perform patch-level classification based on transfer learning. Prediction fusion at both feature and image level is employed for more adaptable performance.

The contributions of this paper can be summarized as follows.

- It proposes a novel lymphoma subtypes classification approach utilizing a multiple hierarchies classification method from local to global, which improves the completeness of features and reduces the spatial complexity of computation simultaneously by combining small patch level feature extraction and efficient image level classification strategies.
- It shows the benefits of appropriate image level classification strategy based on patch level results by comparing three strategies, namely, majority voting, mean score of patches, and weighted sum of patch scores.
- It proposes a novel distance matrix weighting (*DMW*) method to calculate appropriate weights for patch level results and presents improved performance.
- It presents that more effective features can be extracted from transformed image space. Different image space transformations are employed to enhance particular image characteristics. Space-specific feature set can be extracted which is better than features extracted from original images.
- It shows that transfer learning technique based on deep CNN network can provide better efficiency for lymphoma classification. Pretrained Google Inception Net is fine-tuned to improve the performance and leave out time-consuming training process simultaneously.

This paper is organized as follows.  Section 2 introduces the dataset used in this study and illustrates the detailed methodology.  Section 3 shows the experimental setup and results.  Section 4 presents the comparison with other existing works and discussions for experiment performances. Finally, the conclusions are provided in Section 5.

## 2. Materials and Methodology

### 2.1. Dataset

In this study, a public dataset named IICBU malignant lymphoma dataset [18] with respect to studies in National Cancer Institute and National Institute on Aging, both in the United States [19, 20], is used. There are 10 cases for each of these three lymphoma types, namely, CLL, FL, and MCL, and in total 30 H&E stained lymphoma histological slides used for this study. A Zeiss Axioscope white light microscope with a 20× objective and a color digital camera AxioCam MR5 are used to obtain these images. Lesion regions in these slides are digitally photographed and reserved without compression in the* tif* format, using RGB color model, with 1388 × 1040 resolution and 24 bits quantization. The final dataset contains 374 images with 113, 139, and 122 regions of CLL, FL, and MCL. In our study, the whole data set is randomly divided into training and testing sets by a ratio of 7:3.

**Figure 1:**
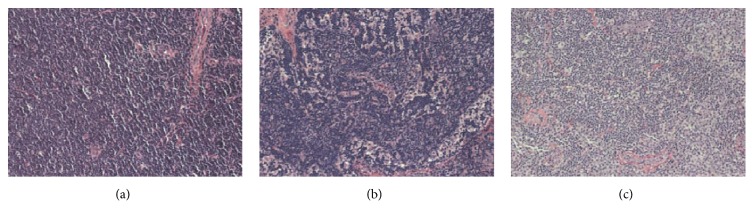
The lymphoma histological image samples for different NHL subtypes: (a) CLL, (b) FL, and (c) MCL.

Figure 1 shows the samples of histological images employed in our experiments to evaluate the proposed approach. A CLL sample is depicted in Figure 1(a), an FL sample is shown in Figure 1(b), and an MCL sample is presented in Figure 1(c). These images are from biopsies of different patients in different hospitals, so the inner class difference could be prominent because of the staining variance [21], which makes the classification task quite challenging.

### 2.2. Method

**Figure 2:**
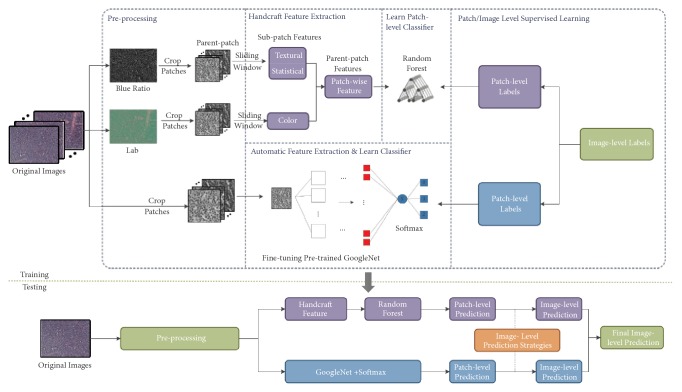
The overall flow of the proposed method.

The proposed classification pipeline takes an H&E stained malignant lymphoma image section as input and finally yields as output the image level classification result with likelihood estimations for each class.  Figure 2 illustrates the overall flow of our proposed methodology which has four basic steps.

*(a) Image Space Transformation*. Both single channel and multiple channel image transformation methods are considered. The original images are mapped into the blue ratio gray space to emphasize cellular morphology. The CIE Lab color space transformation is applied to original RGB images to amplify the color characteristic. These two space transformation methods are used to enhance different features of original images by which targeted discriminative features can be extracted subsequently. This process can also be considered as a preprocessing step. 

*(b) Handcrafted Features Extraction and Classification*. A set of texture and statistical features are obtained from blue ratio gray space and color features are extracted from Lab space images. A hierarchical local-to-global feature extraction method is used to obtain complete patch-wise features in different scales. Then different feature subsets are combined to train a patch level random forest classifier. 

*(c) Transfer Learning Based High-Level Feature Extraction and Classification*. A pretrained deep convolutional neural network is fine-tuned to extract patch level automatic features from cropped original images. A softmax classifier is connected to the output layer to implement patch level classification. 

*(d) Multi-Path Integration and Image Level Classification.* Image level prediction is calculated based on patch level prediction of this image using several strategies. Weighted fusion in both patch level and image level prediction is used to integrate results from different paths.

Detailed description of the proposed pipeline's steps is shown in Figure 2.

#### 2.2.1. Image Space Transformation

**Figure 3:**
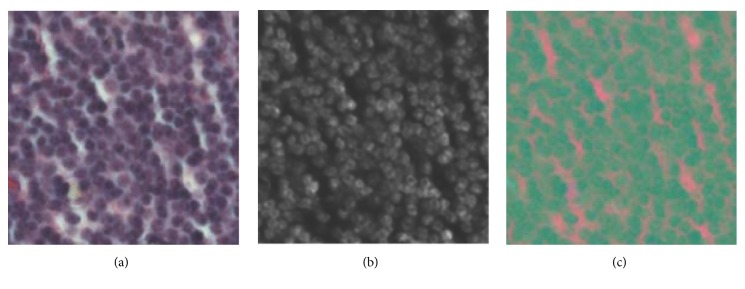
Sample patches of different image spaces. (a) One sample patch of original RGB image for CLL. (b) Blue ratio image. (c) Lab color space image.

*(A) Blue Ratio Image*. Chang* et al.* [22] proposed that RGB images could be transformed into a one channel gray space named blue ratio image to enhance the nuclear dye and attenuate background. And it is time-consuming or even impractical to extract some kinds of handcrafted features in three channel images. Therefore, in order to calculate texture and statistical features of original images in a more efficient way, the original RGB images can be mapped into blue ratio image space. The conversion policy is defined as

(1) $$\begin{array}{c} BR=\frac{100\times B}{1+R+G}\times \frac{256}{1+B+R+G} \end{array}$$

where *B*, *R*, and *G* are intensities of blue, red, and green channels and* BR* represents the intensity at the corresponding position in transformed blue ratio gray space. The first term enhances the nuclear signal and the second term attenuates the background.  Figure 3(b) illustrates the blue ratio transformation result for the original image as shown in Figure 3(a). Subsequently, texture and statistical features are extracted in the transformed gray level images.

*(B) CIE Lab Color Space*. The CIE Lab color space is a color mode enacted by International Commission on Illumination (CIE) in 1976 [16]. It is a device-independent color system based on physiological features. It has three basic coordinates *L∗a∗b∗*, abbreviated as Lab [23].* L* component represents the luminosity, and *a* and *b* represent the green-red and blue-yellow color value components, respectively. The range of* L* channel's value is [0, 100], which corresponds to different lightness from black to white. The range of *a* channel's value is [-128, 127] and* b* channel has values in range [-128, 127].  Figure 3(c) is a sample of Lab color space transformation result for the original image in Figure 3(a).

RGB color space is transformed into XYZ color space first and then the XYZ space is converted into Lab color space [24], as given by (2) and (3), respectively. Subsequently, color features are extracted from the transformed Lab images.

(2) YZ=0.3575800.1804230.2126710.7151600.0721690.0193340.1191930.950227GB

(3) L=116×fYYn−16b=200×fYYn−fZZn

where *X*_*n*_ = 0.950456, *Y*_*n*_ = 1.0, and *Z*_*n*_ = 1.088754. *f*(*t*) is defined as

(4) $$\begin{array}{c} f\left(\;t\;\right)=\left\{\begin{array}{cc} t^{1/3} & if\;\;t>{\left(\frac{6}{29}\right)}^{3}\\ \frac{1}{3}{\left(\frac{29}{6}\right)}^{2}t+\frac{4}{29} & \text{otherwise} \end{array}\right. \end{array}$$

#### 2.2.2. Handcrafted Feature Extraction

*(A) Overall Process*. Based on the image preprocessing results discussed in Section 2.2.1, discriminative patch-wise manual features are extracted in a local to global way. The entire process for manual feature extraction can be expounded in four major steps as follows.

*(a) Cropping Parent Patches*. Big parent patches are cropped in preprocessed 1388 × 1040 images with window size *s*_1_. Parent patches are cropped in a nonoverlapping way to reduce subsequent computation complexity. An appropriate value of parent window size *s*_1_ is necessary to capture local information completely and precisely.

*(b) Cropping Subpatches.* Relatively smaller subpatches are cropped from parent patches using sliding window with window size *s*_2_. In order to fully extract local information, subpatches are cropped in an overlapped way with overlapping ratio *r*_*o*_.

*(c) Extracting Subpatch Features*. The preprocessed single or multiple channel images with original size are first sliced into *s*_1_ × *s*_1_ parent patches. Then *s*_2_ × *s*_2_ subpatches are obtained from each parent patch using sliding window. Specific n-dimensional feature sets are extracted from subpatch. For example, texture features are extracted from single channel patch, and three channel Lab images contribute color features.

*(d) Calculating Parent Patch Features.* Since each parent patch has its corresponding subpatches set, its features can be generated from subpatches' features. As described in Section 2.2.2,* n*-dimensional features are extracted from each subpatch. First, we can calculate the mean and standard deviation as well as the 10th and 90th percentile of each dimension of the* n* features among all subpatches cropped from this parent patch, which yields a total of 4*n*-dimensional features as the parent patch's representation.

Finally, 4*n* features at parent patch level are extracted. Since it is more likely that parent patches contain complete and representative information, computation of features from subpatch level to parent patch level is done to obtain more meaningful and discriminative features. The detailed kinds of original features extracted from subpatch are discussed below.

*(B) Features Extraction in Blue Ratio Images*. As described in Section 2.2.1, blue ratio image emphasizes the foreground in H&E stained histopathology images, namely, the nuclear structure, and cellular morphology is discriminative for tumor classification. In addition, blue ratio image is a single channel gray space image which reduces computation cost of some texture and statistic features.

Local binary pattern (LBP) texture representations and a set of statistical features, expressed as *r*_*LBP*_ and *r*_*sta*_, respectively, as explained below, are extracted from processed blue ratio image in view of its characteristics.

LBP is a local texture descriptor which has the characteristics of simple calculation, insensitivity to illumination changes, and good texture expression ability [25]. The specific operation steps are as follows:

- Set the center pixel of the window with a fixed radius* r* and sampling points' number* p* as the threshold, and compare the pixel values of the* p* sample points with it.
- If the pixel value of the sample point is greater than the center pixel value, the corresponding position of this pixel point is marked as 1, otherwise it is 0.
- The above procedure produces a* p*-bit binary number, which is the LBP value of the center pixel of the window.

Therefore, an LBP operator with *p* sample points in a circular region of radius *r* will produce 2^*p*^ patterns. As the neighborhood sampling points' number *p* increases, the type of binary pattern augments exponentially. The statistical histogram of the LBP patterns is usually used to express image information when the LBP operator is used for texture classification, and excessive pattern types will result in too large data quantity and too sparse histogram. Therefore, it is necessary to perform dimensionality reduction on the original LBP patterns, that is to use fewer feature dimensions to represent image information effectively.

Ojala* et al.* proposed using uniform local binary patterns (ULBP) to reduce the dimension of LBP operator since most LBP patterns only contain two 0-1 or 1-0 transitions in real images [26]. The “uniform pattern” is defined as follows: if the cyclic binary value corresponding to an LBP has at most two 0-1 or 1-0 transitions, this binary pattern is one kind of uniform pattern. Specifically, let* p* be the sampling points' number, there are 2 patterns for 0 transition, 2(*p* − 1) patterns for 1 transition, and (*p* − 1)(*p* − 2) patterns for 2 transitions. Moreover, nonuniform patterns with more than 2 transitions are regarded as one pattern; thus a total of 2 + 2(*p* − 1)+(*p* − 1)(*p* − 2) + 1 = *p*(*p* − 1) + 3 patterns are generated. As a consequence, the statistical histogram dimension of LBP is reduced from 2^*p*^ to *p*(*p* − 1) + 3. For instance, for a fixed radius *r*, when the sampling points number *p* = 8, the feature dimension is reduced from 2^8^ = 256 to 8 × (8 − 1) + 3 = 59.

The detailed LBP and statistical features extraction settings are as follows:

*r*_*LBP*_: ULBP for radii 1 and 2 with 8 sampling points is extracted, which is 118-dimentional for each subpatch. So *r*_*LBP*_ contains 472 features for each parent patch.

*r*_*sta*_: 9 statistical features of the gray level histogram are computed for each subpatch including maximum, minimum, sum, mean, standard deviation, median, first and third quartiles, and interquartile range. So *r*_*sta*_ contains 36 features for each parent patch.

*(C) Features Extraction in Lab Images*. RGB color channels tend to be affected by color variance in images; hence Lab color space is used to extract color features. As illuminated in Section 2.2.1, Lab color space contains three channels of which *a* and *b* channels represent color component. Therefore, the color representation *r*_*Lab*_ is extracted as follows:

*r*_*Lab*_: Extracting statistics from color histogram with 85 bins of* a* and* b* channels, respectively, and cascading the two results. As a result, 170-dimentional features can be extracted from each subpatch and *r*_*Lab*_ contains 680 features for one parent patch.

#### 2.2.3. High-Level Feature Extraction

CNN is a multilayer neural network including convolutional, pooling, and fully connected layers, which can learn features in an automatic and supervised way. It has shown success in biomedical imaging applications [13, 27, 28]. Some customized deep CNN models trained on ImageNet have displayed improved performance, and their effectiveness on biomedical images implies natural and biomedical images which virtually share similar low level features [29].

Since a deep network usually contains a large number of parameters that need to be fully trained and optimized, suitable parameter initialization is extremely helpful for training process, which is of great importance for model's final performance. Poor parameter initialization may lead to slow convergence rate and insufficient parameter training, especially for small biomedical data sets. The deep network trained on ImageNet performs well on the complex classification tasks of other natural images, and some existing studies have shown that classification network learning from natural images has certain transfer effects on biomedical image data sets [30]. This effectiveness, which may originate from the similarity of natural images and biomedical images in some low level texture and shape features, has also been verified by experiments in this paper. Therefore, the pretrained deep network on ImageNet can provide excellent parameter initialization. This paper makes some customized modifications to the network output structure based on the initialized parameters and performs fine-tuning process on the whole network using the lymphoma data set to make it more suitable for our classification task.

Specifically, the Google Inception Net (GoogLeNet) v3 model pretrained on ImageNet [31] is applied to classification task in this study. It is a deep network with 42 layers and provides state-of-the-art classification result for ImageNet dataset. The original RGB image is cropped into *s*_3_ × *s*_3_ patches and 2048-dimensional local features *r*_*G*_ are densely extracted from the last fully connected layer for each patch. The patches are used to perform a basic fine-tuning process, that is, to add a fully connected linear layer with the true number of classes after the last layer in GoogLeNet and perform backpropagation just for last fully connected layers. In this way, the pretrained GoogLeNet is customized to fit characteristics of this specific data set. Finally, a softmax classifier is connected after the network to perform classification. Parameters in other layers keep invariant as in the pretrained model.

By utilizing transferred GoogLeNet, time used for the training process can be saved significantly; meanwhile, the classification performance is improved due to powerful representation ability and high universality of pretrained GoogLeNet.

Note that training a new customized CNN model for specific biomedical imaging application is a more usual choice than using pretrained models. A non-pretrained Google Inception Net v3 implemented by ourselves is experimented for comparison. The model is trained totally using datasets in this study. Experimental result shows the improved efficiency of transfer learning in this dataset with small number of biomedical images.

#### 2.2.4. Integration and Classification

*(A) Patch Level Integration and Classification*. Different feature sets are extracted for each parent patch in Sections 2.2.2 and 2.2.3, including LBP features r_*LBP*_, statistical features *r*_*sta*_, Lab color features *r*_*Lab*_, and high-level features *r*_*G*_. For handcrafted features *r*_*LBP*_, *r*_*sta*_, and *r*_*Lab*_, an auxiliary random forest classifier is trained on specific feature set *F* to perform patch level classification. Feature set *F* can be a single type of manual features, namely, *r*_*LBP*_, *r*_*sta*_, or *r*_*Lab*_, and it can also be a combination of multiple kinds of features, which involves a fusion process at feature level. There are mainly two kinds of feature combination modes considered in this study, which are *F*_1_ : {*r*_*LBP*_, *r*_*sta*_} and *F*_2_ : {*r*_*LBP*_, *r*_*sta*_, *r*_*Lab*_}, since feature set *F*_1_ is extracted from blue ratio image and *F*_2_ is an expansion of *F*_1_ with additional color feature from Lab image. For high-level feature *r*_*G*_, the softmax classifier cascaded after GoogLeNet is used for patch level classification.

*(B) Image Level Classification*. One original sized image can be cropped into a set of parent patches. There are different methods for parent patch classification as discussed in Sections 2.2.2, 2.2.3, and 2.2.4. For each parent patch, the probability scores for three classes can be calculated. Image level classification can be performed based on parent patch prediction result considering that patches of the corresponding original image reflect local knowledge of the whole image. For image level classification, three strategies are chosen to make sufficient use of patch level result and to obtain convictive image level prediction. Two of them are commonly used approaches illustrated as follows. 

*(a) Majority Voting (MV)*. Assume an original image* I* can be cropped into* m* parent patches. Let *m*_1_, *m*_2_, and *m*_3_ denote the numbers of patches classified as the first, second, and third classes. *K*(max⁡([*m*_1_, *m*_2_, *m*_3_]), [*m*_1_, *m*_2_, *m*_3_]) calculates the final prediction class of the whole image, where *K*(*x*, *y*) is the function that calculates value *x*'s index in array *y*.

*(b) Mean Score (MS) of All Patches*. Since each parent patch has probability scores for three classes, scores for the whole image can be calculated taking average over all parent patches' scores. The image level classification result can be obtained based on image scores.

In addition, a novel strategy to calculate weighted sum of patch scores for image level classification is proposed. Different patches are corresponding to different locations of one image and contain various local information, so they own different discrimination and contribution for classification task. Therefore, appropriate weights for different patches are favorable for acquisition of conclusive image classification result. The most crucial part is the determination of weights. A distance matrix weighting (*DMW*) method is proposed to determine weights in this study, which is described below.

Suppose the patch with closer association to other patches is inclined to provide more contribution to the whole image. This hypothesis is made based on the fact that the lymphoma samples in this study are all sections from lesions, so class-specific characteristics are reflected on most cropped patches.

One original image* I* can be cropped into* m* parent patches {*I*_1_, *I*_2_,…, *I*_*m*_}. Specific feature set can be extracted for each parent patch, which generates* m* patch-wise feature sets {*r*_1_, *r*_2_,…, *r*_*m*_}. And patch level prediction scores set {*s*_1_, *s*_2_,…, *s*_*m*_} can be obtained from the trained classifier based on feature set for each parent patch, where *s*_*i*_ is the classification score for the *i*^*th*^ patch and contains three score values corresponding to three classes. For one patch *I*_*k*_, a weight *w*_*k*_ should be calculated.

First, a distance matrix *M*_*d*_ is generated for all patches based on their feature values. Let *d*_*ij*_ = *D*(*r*_*i*_, *r*_*j*_) denote the element of row *i* and column *j* in *M*_*d*_, where *D*(*x*, *y*) is a function to calculate distance between vectors *x* and *y*, and Euclidean distance is used in our study. Then the average distance *da*_*k*_ between parent patch *I*_*k*_ and all others is calculated based on *M*_*d*_ as follows:

(5) $$\begin{array}{c} da_{k}=\frac{1}{m-1}\overset{m}{\underset{i=1}{\sum }}d_{ki} \end{array}$$

In essence, *da*_*k*_ is the mean value of the *k*^*th*^ row in *M*_*d*_, and the patch with smaller average distance should be given greater weights. Thus a distance score *Sd*_*k*_ for patch *I*_*k*_ is defined by taking the reciprocal of *da*_*k*_ to represent this inversely proportional relationship, making it convenient to calculate patch weight.

(6) $$\begin{array}{c} Sd_{k}=\frac{1}{da_{k}}=\left(\;m-1\;\right)\frac{1}{\sum_{i=1}^{m}d_{ki}} \end{array}$$

Then the weight *w*_*k*_ for patch *I*_*k*_ is calculated as follows:

(7) $$\begin{array}{c} w_{k}=Sd_{k}\frac{1}{\sum_{i=1}^{m}Sd_{i}} \end{array}$$

Finally the image-level classification score *S*_*I*_ is obtained based on patch level prediction scores set *S*{*s*_1_, *s*_2_,…, *s*_*m*_} and weights set *W*{*w*_1_, *w*_2_,…, *w*_*m*_} as shown in the following equation:

(8) $$\begin{array}{c} S_{I}=\overset{m}{\underset{i=1}{\sum }}w_{i}\cdot s_{i} \end{array}$$

As a result, we consider totally three image-level classification strategies denoted as* MV*,* MS,* and* DMW*.

*(C) Image Level Integration*. Different feature set selection methods at patch level can be combined with one of the image level classification strategies. The probability scores of multiple methods can be fused at both patch level and image level. There are mainly five method combination modes experimentalized in this study:

- *C*_1_(*F*_1_ + *DMW*): Feature set *F*_1_ at patch level combined with* DMW* at image level.
- *C*_2_(*F*_2_ + *MS*): Feature set *F*_2_ at patch level combined with* MS* method at image level.
- *C*_3_(*F*_1_ + *F*_2_ + *DMW*): For one original image, two patch level prediction scores sets *S*_1_ = {*s*_1_^(1)^, *s*_2_^(1)^,…, *s*_*m*_^(1)^}, *S*_2_ = {*s*_1_^(2)^, *s*_2_^(2)^,…, *s*_*m*_^(2)^} of cropped parent patches can be obtained based on *F*_1_ and *F*_2_, respectively, where *s*_*i*_^(*j*)^ is the classification score of the image's *i*^*th*^ parent patch based on feature set *F*_*j*_. Score fusion at patch level can be performed by calculating weighted sum of *S*_1_ and *S*_2_ to integrate characteristics of two methods. Then the final patch level probability score set *S* = *w*_*P*_ · *S*_1_ + (1 − *w*_*P*_) · *S*_2_ can be obtained.* DMW* strategy is used for image level classification.
- *C*_4_(*r*_*G*_ + *DMW*): High-level feature set *r*_*G*_ combined with* DMW* at image level.
- *C*_5_(*C*_3_ + *C*_4_): Weighted summation with weight *w*_*I*_ of image level results *S*_*I*3_ and *S*_*I*4_ from *C*_3_ and *C*_4_. The final image level probability score *S*_*I*5_ = *w*_*I*_ · *S*_*I*3_ + (1 − *w*_*I*_) · *S*_*I*4_.

Method combination modes *C*_1_, *C*_2_, and *C*_3_ are based on handcrafted features among which *C*_3_ integrates features in *C*_1_ and *C*_2_, whereas *C*_4_ is totally based on deep automatic features. In order to bind handcrafted and automatic features, the image level probability scores of *C*_3_ and *C*_4_ are fused by weighted summation in *C*_5_.

Note that there are more alternative combination modes at features, patches, and images levels. The core principle is to choose different typical feature sets in different combination modes, and three image-level strategies are all experimentally evaluated with specific features and the best strategy is finally combined with this feature set (except image level fusion method *C*_5_). Finally, only several typical choices are experimentally compared to discuss each part's classification contribution.

## 3. Experiments and Results

### 3.1. Evaluation Metrics

Classification performances at both patch level and image level can be evaluated using several commonly used metrics calculated as follows:

(9) ACC=1n∑i=1nIfxi=yi

(10) SENci=NumPCciNumGTci

(11) SPEci=NumPC−ciNumGT−ci

(12) Pci=NumPCciNumPTci

(13) Rci=NumPCciNumGTci

(14) F1ci=2×Pci×RciPci+Rci

where ACC represents the overall classification accuracy. SEN(*c*_*i*_) and SPE(*c*_*i*_) mean the sensitivity and specificity for class *c*_*i*_. P(*c*_*i*_), R(*c*_*i*_), and F_1_(*c*_*i*_) are precision, recall, and *F*_1_ score for class *c*_*i*_, respectively. I(*f*(*x*_*i*_) = *y*_*i*_) defines that if *f*(*x*_*i*_) = *y*_*i*_, I(*f*(*x*_*i*_) = *y*_*i*_) = 1, otherwise I(*f*(*x*_*i*_) = *y*_*i*_) = 0. −*c*_*i*_ denotes all the other classes except *c*_*i*_. Num(PC(*c*_*i*_)), Num(PT(*c*_*i*_)), and Num(GT(*c*_*i*_)) represent correctly predicted number of class *c*_*i*_, the total number of all predicted *c*_*i*_, and the total number of *c*_*i*_ in the ground truth, respectively. Note that recall is equal to sensitivity. Furthermore, area under the receiver operating characteristic curve (AUC) is also calculated for evaluation.

### 3.2. Parameter Sensitivity

**Figure 4:**
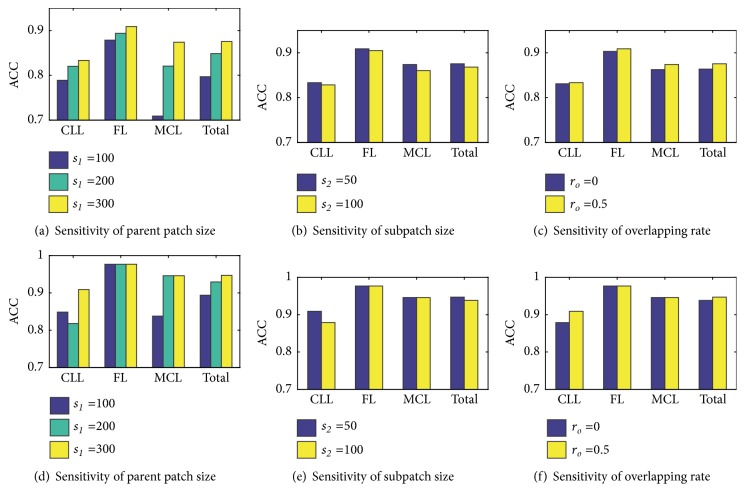
Sensitivity for three parameters at both patch and image levels.

Some parameters in the study have high sensitivity for final performances, such as sizes of parent and subpatches, overlapping rate of subpatches, number of decision trees of random forest classifier, and fine-tuning iteration times of pretrained GoogLeNet. Sensitivity of these parameters is experimentally analyzed by observing both patch and corresponding image levels classification performances when just one primary parameter is adjusted at a time, as shown in Figure 4. Sensitivity for three parameters: parent patch size *s*_1_, subpatch size *s*_2_, and subpatch overlap rate *r*_*o*_ are depicted in Figure 4. (a), (b), (c) are patch level accuracy and (d), (e), (f) are the corresponding image level accuracy.

Figures 4(a) and 4(d) show the patch and image levels accuracy with different parent patch size *s*_1_, respectively. Let the subpatch size *s*_2_ = 50 and its overlapping rate *r*_*o*_ = 0.5. The selected feature set is *r*_*LBP*_ + *r*_*sta*_ extracted from blue ratio image and a random forest classifier with 350 decision trees is trained for classification. Majority voting (*MV*) is applied for image level prediction. Note that the above is the default experimental setup for all the subsequent experiments. Performances for three parent patch scales (100×100, 200×200, and 300×300) are compared at both patch level and image level.

Subpatch size impacts completeness of local knowledge extraction and then may make a difference for the final results. The accuracy for different subpatch size at patch and image levels is shown in Figures 4(b) and 4(e), respectively. Parent patch is cropped in a nonoverlapping way considering calculation complexity, so only the subpatch overlapping rate *r*_*o*_ is experimented as shown in Figures 4(c) and 4(f). Parent patch size is fixed to *s*_1_ = 300 in above experiments. Default setup is used for other parameters.

**Figure 5:**
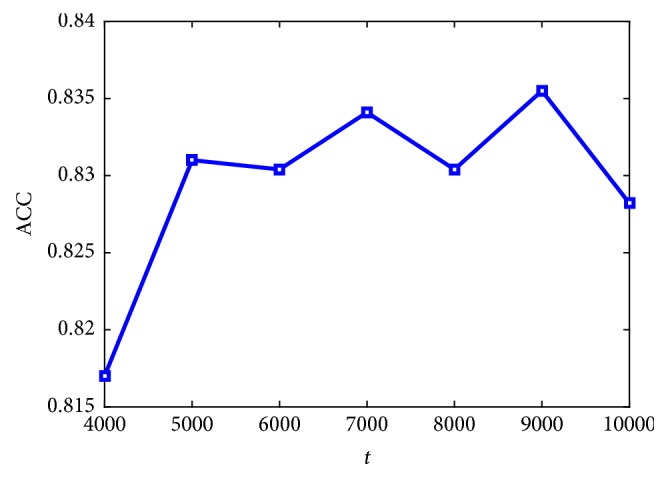
Patch level classification accuracy of transferred GoogLeNet with different fine-tuning iteration times* t*.

Transfer learning is used by applying the pretrained GoogLeNet model and performing fine-tuning process for the last fully connected layer using dataset in this study. Patch-level accuracy on testing set for different iteration times *t* in fine-tuning process is shown in Figure 5. Size *s*_3_ of the input original RGB patch is set to 300. Ten percent of training data is randomly divided into validation set to monitor training situation. The model is implemented based on tensorflow. Learning rate *e* = 0.001 and cross entropy loss function are used. Stochastic gradient descent (SGD) is selected as the optimizer and the training batch size is set to 100. As shown in Figure 5, the test accuracy increases as *t* increases and reaches maximum 83.55% when *t*=9000, but after that, the accuracy begins to decline because of overfitting.

**Figure 6:**
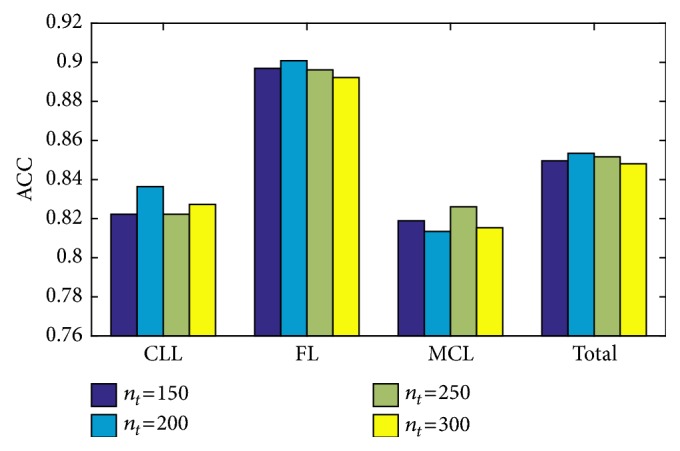
Patch level classification accuracy for different decision tree number.

A random forest (RF) classifier is used for patch-wise classification based on handcrafted features as discussed in Section 2.2.4. The influence of RF's decision tree number *n*_*t*_ is experimentally evaluated.  Figure 6 exhibits patch level classification accuracy with different decision tree numbers. Parent patch size *s*_1_ is set to 200. Other related parameters are determined using default setup. The effect of decision tree number varies among different classes; however, as a whole, RF classifier obtains best patch level classification result when *n*_*t*_ = 200. Note that Figure 6 only shows patch level performance, and rational choice for image level classification strategy may make additional adjustment for final prediction.

Weighted fusion is used at both patch and image levels as described in Section 2.2.4 with weights *w*_*P*_ and *w*_*I*_, which are set to 0.7 and 0.5, respectively, using parameter scanning.

### 3.3. Experimental Results

To evaluate the contributions of different feature sets, several kinds of features involved in this study are tested and compared. Specific RF classifiers are trained for different handcrafted feature sets *r*_*LBP*_, *r*_*sta*_, *r*_*sta*_ as well as their combination. Fine-tuned GoogLeNet combined with softmax is used to test high-level feature *r*_*G*_. To confirm the efficiency of the concerned features, two additional kinds of handcrafted features Gist [32] and local phase quantization (LPQ) [33] are considered. The accuracy of different feature sets is displayed in Table 1, where parent patch size *s*_1_ = 200. Fine-tuning iteration time of GoogLeNet is set to 9000.

**Figure 7:**
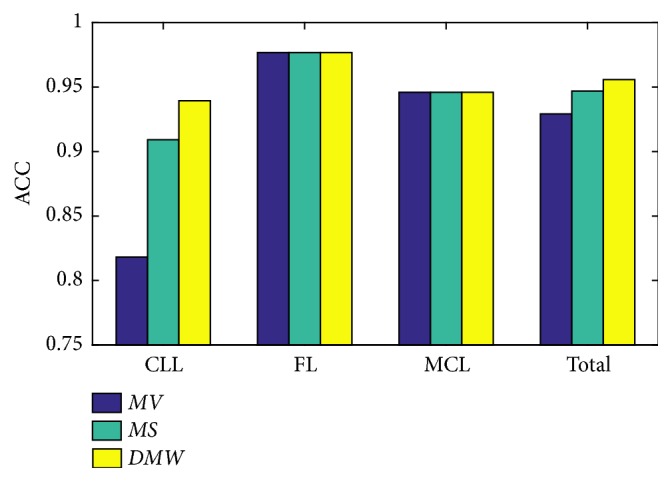
Classification accuracy of different image level classification strategies.

Several strategies in image level classification are used to take full advantages of patch level results as illustrated in Section 2.2.4.  Figure 7 shows the image level accuracy of the three strategies. Parent patch size *s*_1_ = 200, and default setup is used for other parameters. The impact of different image level strategies is mainly reflected in classification performance for CLL in this setup. By utilizing the proposed* DMW* image level strategy, the accuracy for CLL is significantly improved compared with commonly used* MV*. In fact, our experimental result shows the proposed method* DMW* can provide better or comparative performance for different parameters setups, which indicates its efficiency is superior to those of other commonly used methods to calculate image level results based on patch level predictions.

**Figure 8:**
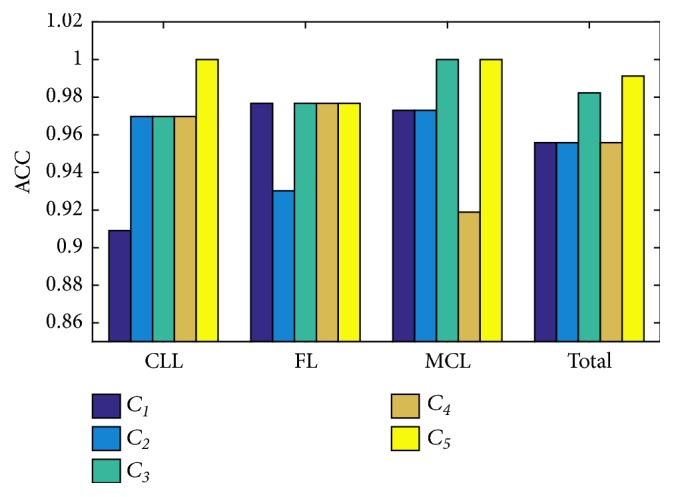
Image level classification accuracy for different method combination modes.

There are mainly several method combination modes (a)~(e) in Section 2.2.4. Fusion at patch level, namely, combination mode *C*_3_, is based on *C*_1_ and *C*_2_. Fusion at image level is implemented in *C*_5_ based on *C*_3_ and *C*_4_. Image level classification accuracy of the five combination schemes *C*_1_ ~ *C*_5_ is shown in Figure 8. Performances for *C*_1_, *C*_2_, and *C*_4_ are relatively low because these combination modes are based on specific feature set, but they show different discrimination power for three classes. Therefore, combination modes that fuse their characteristics obtain improved performances. *C*_3_ combines *C*_1_ and *C*_2_ by score fusion at patch level which provides prominent performance improvement. *C*_5_ integrates *C*_3_ and *C*_4_ through weighted summation at image level results and obtains further amelioration for classification of CLL, which is considered as the final scheme for this classification task.  Table 2 shows the classification indices including overall accuracy (ACC), overall area under the curve (AUC) and AUC, sensitivity (SEN), recall (R), specificity (SPE), precision (P), and *F*_1_ score for each class of different method combination modes *C*_1_ ~ *C*_5_.

## 4. Comparison and Discussions

To further evaluate our model, several existing methods for this lymphoma dataset are used for comparison, as shown in Table 3. Method combination mode *C*_5_ is used as a representative of our method since it has the best performance and shows better classification results than those of the state-of-the-art methods can be obtained. The current state-of-the-art method [15] proposed Fisher vector (FV) encoding combined with multiple types of local features. It applied a novel separation-guided dimension reduction method and provides 97.9% accuracy and 0.993 AUC. Codella* et al.* [14] used descriptors including LBP and CNN based on segmented results. SVM was used as classifier and it obtained 95.5% accuracy. Song* et al.* [8] proposed a method to extract high-dimensional descriptor and subcategory discriminant transform (SDT) was used to enhance discriminative power of descriptors, which achieved the accuracy of 96.8%. Meng* et al.* [7] proposed a framework based on the Collateral Representative Subspace Projection Modeling (CRSPM) supervised classification model for histology image classification. Shamir* et al.* [17] presented an open source utility Wndchrm for standardized classification which provided accuracy of 85% for lymphoma classification. In our study, we achieve 99.1% accuracy and 0.998 AUC with method combination *C*_5_.

Table 3 provides a quantitative comparison in terms of AUC and ACC values, which can be seen as complementary. The results demonstrate the advantages of our proposed model for lymphoma classification over the state-of-the-art approaches. Song* et al.* [15] calculated both ACC and AUC and achieved the best performance in existing approaches with 97.9% ACC, while we achieve 99.1% accuracy employing the *C*_5_ combination mode with a similar AUC value. This suggests that the extracted feature sets and RF classifier in our study is more discriminative for malignant lymphoma pathological images.

According to Table 3, our method achieves better accuracy than all other existing methods. Shamir* et al.* [17] proposed solutions based on an open source platform for standardized support of different problems, including tissue age differentiation, subcellular protein localization, lymphoma subtyping, and pollen grain distinction. It is not targeted so the performance for lymphoma is relatively low. Meng* et al.* [7] and Song* et al.* [8] extracted high-dimensional features and applied popular machine learning method; however, they have not considered deep learning techniques. According to our research, high-level features of CNN have promising contribution for final classification. Codella* et al.* [14] and Song* et al.* [15] both combined handcrafted and high-level features to perform classification. Codella* et al.* [14] applied a segmentation process which is time-consuming, and the segmentation-based features can easily be nondiscriminative due to imprecise segment results. Song* et al.* [15] utilized Fisher vector to encode the whole image, which resulted in low efficiency in feature extraction stage. In addition, Codella* et al.* [14] and Song* et al.* [15] performed classification with SVM, while our study experimentally confirmed that random forest classifier can achieve better performance for this classification task.

To sum up, accurate lymphoma classification is an important prerequisite for malignant lymphoma CADs. Our proposed classification method performs well in different image space transformations of original images, noting that blue ratio and Lab space amplify the texture and color characteristics of original images, respectively. Therefore, the subsequent feature extraction with customized feature sets specific to image spaces is meaningful. Classification is performed utilizing multiple hierarchies of local knowledge, which ensures the completeness of features and efficiency of the whole approach. Patch based classification reduces the spatial complexity but sacrifices some local information, so the strategy for image level classification is investigated in detail for a remedy. Transfer learning is used to reduce consumption of time and it shows improved performance. Multiple paths are combined at different stages to fuse their characteristics sufficiently. Random forest classifier is employed, and it shows better performances than commonly used SVM.

## 5. Conclusions

This paper proposes a new CAD model for lymphoma classification on H&E stained histopathological images. This model includes three major steps, namely, image space transformation, feature extraction, and classification design. First, original images are transformed into different image spaces including blue ratio and Lab. Then two hierarchies of patches are cropped to perform a local-to-global classification. After that, specific feature set is extracted from corresponding image space. RF classifier is then trained for patch level classification based on handcrafted features. Meanwhile, transfer learning is introduced by utilization of a pretrained GoogLeNet model which also performs patch level classification. Finally, image level classification strategies are discussed and fusion at different levels is performed. The identification task is performed on IICBU Malignant Lymphoma Dataset. The experiment results and comparisons with the related work present that out proposed model can achieve better identification performances than others. We demonstrate the benefits of our multihierarchy classification scheme and the novel* DMW* method for weights computation of patch-level results, as well as the advantages of image space transformation.

In the future, we intend to apply the proposed model on more kinds of datasets to evaluate its generality when extended to different problems. The analysis of alternative for pretrained CNN can be performed to explore the potential of deep learning. Moreover, since the lymphoma sections in this study are all from lesions, a preceding lesion identification procedure can be designed for those datasets containing extra normal tissues. Then the proposed hierarchical strategy can be applied to the classification for precise identified lesions.

## Figures and Tables

**Table 1:** Patch level classification accuracy for different feature set.

Feature Set	CLL	FL	MCL	Total
Gist	0.5424	0.5829	0.6252	0.5850
LPQ	0.6556	0.8070	0.6405	0.7083
*r*_*LBP*_	0.7343	0.8171	0.7000	0.7546
*r*_*sta*_	0.7828	0.8713	0.7414	0.8029
*r*_*Lab*_	0.7394	0.8837	0.8631	0.8348
*r*_*G*_	0.7394	0.8837	0.8631	0.8355
{*r*_*LBP*_, *r*_*sta*_}	0.8253	0.8969	0.8324	0.8549
{*r*_*LBP*_, *r*_*sta*_, *r*_*Lab*_}	0.9283	0.9217	0.9324	0.9271

**Table 2:** Image level classification indices for different method combination modes.

Index		*C*_1_	*C*_2_	*C*_3_	*C*_4_	*C*_5_
ACC	Overall	0.9558	0.9558	0.9823	0.9558	0.9912

AUC	Overall	0.9846	0.9971	0.9943	0.9894	0.9982
	CLL	0.9709	0.9903	0.9820	0.9875	0.9972
	FL	0.9987	1	1	0.9907	0.9960
	MCL	0.9971	0.9985	1	0.9912	1

SEN/R	CLL	0.9091	0.9697	0.9697	0.9697	1
	FL	0.9767	0.9302	0.9767	0.9429	0.9767
	MCL	0.9730	0.9730	1	0.9189	1

SPE	CLL	0.9750	0.9500	0.9875	0.9500	0.9875
	FL	0.9429	0.9714	0.9857	0.9429	1
	MCL	0.9474	0.9474	0.9737	0.9737	0.9868

P	CLL	0.9677	0.8889	0.9697	0.9412	0.9706
	FL	0.9767	1	1	0.9545	1
	MCL	0.9231	0.9730	0.9737	0.9414	1

F_1_	CLL	0.9375	0.9275	0.9697	0.9552	0.9851
	FL	0.9767	0.9639	0.9882	0.9655	0.9882
	MCL	0.9474	0.9730	0.9867	0.9444	1

**Table 3:** Image level classification performance of our method and existing studies.

Index	Ours	[15]	[17]	[7]	[14]	[8]
ACC(%)	99.1	97.9	85	92.7	95.5	96.8

AUC	0.998	0.993	-	-	-	-

## Data Availability

(1) The dataset includes three types of malignant lymphoma. (2) The data can be accessed from https://ome.grc.nia.nih.gov/iicbu2008/lymphoma/index.html. (3) In addition, there are no restrictions on data access.
